# Regulation of dental consultations in primary health care and performance of services in dental speciality centers

**DOI:** 10.1186/s12913-023-09597-z

**Published:** 2023-06-09

**Authors:** Bárbara Campos da Silva, Giovana Soares Buzinaro, Jhenyffer Andrade Viana Cabral, Inara Pereira da Cunha, Valéria Rodrigues de Lacerda, Rafael Aiello Bomfim

**Affiliations:** 1 School of Dentistry, Federal University of Mato Grosso do Sul, Campo Grande, Brazil; 2 Escola de Saúde Pública, Escola de Saúde Pública de Mato Grosso do Sul, Campo Grande, Mato Grosso do Sul, Brazil

**Keywords:** Referral and consultation, Dental Specialties, Social participation

## Abstract

**Objective:**

This study analyzed the regulation of dental specialty centers (CEOs) coordinated exclusively by Primary Health Care (PHC) in four primary outcomes: access and dental consultation, reception services, bonding and responsibility, and social participation.

**Methods:**

A cross-sectional study was carried out using secondary data from the National Program for the Improvement of Access and Quality of Dental Specialty Centers (PMAQ-CEO): second cycle, using multilevel logistic regression to calculate the odds ratio (OR) and individual covariates.

**Results:**

The analytical sample consisted of 9,599 CEO users who had completed all the variables analyzed. Of these, 63.5% were referred to the CEO by PHC. Dental care regulated by PHC was related to better access (OR 1.36, CI 95% 1.10–1.68), better reception (OR 1.33, CI 95% 1.03–1.71), better bonding and responsibility (OR 1.36, CI 95% 0.91–2.04), and social participation (OR 1.13, CI 95% 0.93–1.35) compared to those not regulated by primary health care as the exclusive pathway.

**Conclusion:**

The regulation of access to the CEO coordinated by PHC presented the best performance. It is suggested that this form of PHC regulation, as a route for dental specialty centers, can be established in the national oral health care policy for better service performance.

## Introduction

The Dental Specialty Centers (CEO) are responsible for offering specialized procedures for Brazilian citizens, such as complex dental surgeries, dental prostheses, endodontic treatment, and complex periodontal procedures within the Oral Health Care Network. They work in line with Primary Health Care, ensuring the interface with the health system according to the city’s epidemiological reality [1] and patient needs. The main problems in the functioning of patient regulation in CEOs are the absence of protocols that guide professionals on when and how to refer the patient, the high rate of absenteeism and evasion, and the large number of people who seek dental care directly in CEOs, without going to the PHC before [2–5].

The PMAQ-CEO, part of the National Program for the Improvement of Access and Quality (PMAQ), measures access to dental care, reception, bond and responsibility, and social participation for the positive perception of the patient about care in PHC and CEOs [6, 7]. Furthermore, it aims to collect information from CEOs to stimulate care and education practices, strengthening qualification actions to generate improvements in the services provided to the population [8]. Its first phase consists of the commitment between professionals and managers with the Ministry of Health, the second consists of external evaluations, and the third phase consists of the commitment of professionals with the federative units so that there is a cycle of affirmation of the results achieved by the members of the PMAQ-CEO. In the second cycle, external evaluations carried out with users allow us to understand indicators of specialized care, supporting their planning [9]. Using the tool, it is possible to classify the performance of these centers and identify the format of regulation of services within the Unified Health System (SUS) [10, 11].

In the provision of specialized care, few reach the minimum goals of the Ministry of Health [12] and only 57.2% have access to care according to the reference demand [2]. As primary health care (PHC) is the path for citizens and coordinators of comprehensive vertical care, it is possible to identify the difficulties related to ensuring integration between care levels [13, 14]. Understanding the relevance of user monitoring and evaluation actions to this integration can enable a better understanding of the service offered [2].

In this way, this study seeks to analyze, through the data collected by the PMAQ-CEO, whether the regulation of CEOs consultation only by PHC is understood by the user as better service performance, allowing better care management. The hypothesis to be tested is that when patients are referred to CEOs by primary health care, all performance dimensions are better compared to accesses by other ways.

## Methods

This was a cross-sectional study that used data from the second cycle of the PMAQ-CEO, which occurred in the years 2017 and 2018. For this, questionnaires were prepared by the General Coordination of Oral Health of the Ministry of Health and with Teaching and Research Institutions. The program was organized in three phases and comprised a continuous cycle of improving the access and quality of CEOs, namely: (1) Adhesion and Hiring; (2) Certification, and (3) Recontracting. The development axis is organized into five dimensions: Self-assessment; Indicator Monitoring; Permanent Education; Institutional Support; and cooperation [15].

The adhesion and contractualization phase, called phase I, consisted of the indication by the municipal CEOs and state managers through the PMAQ-CEO system, with the Federal District being responsible for confirming adhesion and contracting [15]. The External Evaluation of the 2nd cycle of the PMAQ-CEO, which constitutes the second phase of the program cycle, was aimed at evaluating specialized services of oral health. The questionnaires were applied by external state evaluators previously selected and trained by the Ministry of Health, through coordination teams and professors from federal institutions, who used a tablet with the instrument application for data collection. The external assessors observed the structural characteristics, availability of equipment, materials, supplies, instruments, and five elements related to the organization of the work process, verified through interviews with the service manager and health professionals. In Phase III, interviews were conducted with ten users of each CEO who participated in the investigation, with 10,420 interviews related to (1) access and dental consultation; (2) reception services; (3) bond and responsibility; and (4) participation/social control [15].

The data obtained from the three PMAQ-CEO modules were exported to the Microsoft Office Excel 2010 program. Then they were imported into Stata v.14 (Stata, College Station, TX). Through the MERGE procedure, the worksheets referring to the interviews were linked to three phases (phases II and I to III) and variables associated with the number of health services (National Registry of Health Establishments). This procedure allows for information at the individual level (level 1) and on the provision of services (level 2) [16]. The final sample consisted of 1,042 CEOs, since 55 were excluded from PMAQ-CEO because they were closed, under renovation, discredited by the Ministry of Health or refused to participate in the second cycle of the Access and Quality Improvement Program for Dental Specialty Centers (PMAQ-CEO). Participants with incomplete variables or who were seen on the first day at the CEO were excluded from the survey.

The study included users over 18 years of age who were present on the day of the research. PMAQ-CEO was performed following the standards required by the Declaration of Helsinki and approved by the Research Ethics Council under protocol 23458213.0.1001.5208. All participants received and signed the Free and Informed Consent Form in two copies.

### Main exposure (regulation by the primary health care)

The main exposure was the regulation of consultations at the service level. To create this variable, the following question was used for the unit manager: What is (are) the possible way(s) to schedule the consultation for the minimum specialities of the CEO? It was possible to check more than one option by the manager.

1) Is the appointment scheduled by the Basic Health Unit? (Yes/No)

2) The consultation is scheduled by the patient at the specialized consultation regulation centre (yes / no).

3) The appointment is scheduled by the patient who receives the form/referral from the Basic Health Unit (yes/no).

4) The appointment is scheduled by the patient directly with the CEO, without going through primary care (yes / no).

5) There is no defined path (yes / no).

In this way, the regulation variable was created considering whether the individual goes through the referral and counter-referral system, that is, whether they are referred to the CEO by the PHC. In this sense, the yes answers to questions 1 and 3 were considered correctly regulated. However, if they answered yes to questions 2, 4, and 5, they were considered not regulated by primary care, regardless of having answered yes to questions 1 and 3, because in the service, there is the option of scheduling an appointment that is not through the door of entry into the SUS, which is primary health care.

### Dependent variables

In the present study, the four domains related to service performance were considered, according to a previous study that analyzed the performance of dental specialty services related to racial inequalities [16].

#### Access

Regarding the dimension of access and dental consultation, the five outcomes were: 1) Was the consultation at a defined time? Yes (1) and no (0); two). Do they ask what is the best time for the consultation? Yes (1) and no (0); 3) Does the consultation occur in less than a week? Yes (1) and no (0); 4) Do the opening hours suit your needs? Yes (1) and no (0) and 5) Time to get to dental specialty centers for less than 1 h? Yes (1) and No (0).

#### Reception

Concerning the dimension of reception services, the two results obtained were: (1) Is the reception good / very good? Yes (1) and no (0); (2) Was the reception always respectful? Always (1), not always (0).

#### Bonding and responsibility

Regarding the bond and responsibility dimension, the three outcomes were: (1) During treatment, how often do professionals advise you to recover, such as the need for rest, adequate food, use of medications, etc.? Always (1) not always (0); (2) when you need to ask questions after the consultation, do you find it easy to talk to the professionals who attended to you? Always (1) and not always (0); (3) In general, good/very good service? Yes (1) and no (0).

#### Social participation

Regarding the participation/social control dimension, the two outcomes were: (1) Did you know the Ombudsman? Yes (1) and no (0), and; (2) It is easy to make a complaint/suggestion? Yes (1) and no (0).

In the four dimensions, a score was created by the sum of all questions analyzed [16]. The same performance variables were them dichotomized for each dimension considering the median (points below the median and equal to or above the median) as some elements were correlated with each other in each dimension (p < 0.05). For example, in the access dimension, each of the five questions was added and divided by the number of questions, to have a access dimension score for each repondent. So, after creating this score, we created a cutoff point referring to the median point of all scores, and scores equal or above the median point were considered a good reception, entering the value one and below the median point considered a bad reception, entering the value 0 (zero), to enter the regression models carried out. The same was done with all analyzed dimensions. For more information on the calculation of service performance dimensions, they can be consulted (Bomfim et al., 2022) [16].

### Covariates

The covariates were sex (male, female), age (divided between groups 18–34, 35–44, 45–59 and 60 + years), schooling in years (0–8, and 8 + years of formal education) and home coverage by the Family Health Strategy (FHS) (yes/no).

### Statistical analysis

A logistic regression model was estimated to investigate the regulation of dental specialty centers by the Primary Health Care(PHC), adjusted for education, sex, age of participants, and FHS coverage. Associations between regulation and main outcomes were estimated using multilevel logistic regression and expressed as odds ratios (OR), after evaluating for Akaike information criteria (AIC) and Bayesian information criteria (BIC). The individual variables were at level 1 and the contextual variable of regulation at level 2. This model presented the adjusted coefficients of our main exposure (regulation by PHC). All analyzes were performed on STATA 14.2 (College Station, TX, USA).

## Results

Among the individuals interviewed and with complete data, 61.2% were women, 47.3% self-declared brown, 57.5% had more than 8 years of formal education, and 34.2% were between 18 and 34 years of age. Of these, 88.5% reported taking less than an hour to get to the CEO to whom they were referred, but in 52.5% of the cases there was no appointment, and appointments were scheduled more than a week in advance in 69% of the cases. cases and 96.2% of users reported meeting expectations, although in general they were not asked if the available time was the best for the patient(Table 1).

**Table 1:** Descriptive characteristics of users. The PMAQ-CEO 2018-19 study (n = 9599)

**Individual Variables**	**N = 9,599**	**%**	
**Race Group**			
Whites	3451	35,9	
Browns	4540	47,3	
Blacks	1293	13,5	
Asian	206	2,2	
Indigenous	109	1,1	
**Gender**			
Female	6587	61.2	
Male	3012	31.4	
**Schooling**			
0 to 8 years	4416	42,5	
More than 8 years	5975	57,5	
**Age**			
18–34	3285	34,2	
35–44	2245	23,4	
45–59	2647	27,6	
> 60 years	1422	14,8	
**Outcomes**			
**Access**			
**Less than 1 h to arrive?**			
Yes	8493	88.5	
No	1106	11.5	
**Scheduled attendance?**			
Yes	4563	47.5	
No	5036	52.5	
**Asked about the best time?**			
Yes	3176	33.1	
No	6423	66.9	
**Less than 1 week of dental attendance?**			
Yes	2980	31.0	
No	6619	69.0	
**Opening hours met expectations?**			
Yes	9238	96.2	
No	361	3.8	
**Reception**			
**Reception good?**			
Yes	9192	95.8	
No	407	4.2	
**Always respectful?**			
Yes	9340	97.3	
No	259	2.7	
**Bond and Responsabilization**			
**Oriented during treatment?**			
Yes	7885	82.1	
No	1714	17.9	
**Observed after treatment?**			
Yes	8776	91.4	
No	823	8.6	
**Good attendance?**			
Yes	9115	95.0	
No	484	5.0	
**Social participation**			
**Knows the ombudsman?**			
Yes	3796	39.6	
No	5803	60.5	
**Is it easy to complain?**			
Yes	8961	93.3	
No	638	6.7	
**Contextual**			
**Attendance regulated by PHC?**			
Yes	6096	63,5	
No	3502	36,5	

The reception and service of the specialty centers were considered good/very good and always respectful services; patients were generally oriented during care or did not feel the need for clarification. The absolute majority of users claimed to not be aware of the existence of the Ombudsman and stated that they did not need to make a complaint or that, when needed, the service was easily accessible. Of the total number of people interviewed with complete data on all variables analyzed (n = 9,599), 63.5% were referred by Primary Health Care, where they were treated in primary care at registered dental specialty centers.

The results presented in Table 2 reveal that the regulation of referrals to dental specialty centers by primary care is related to ease of access (OR 1.36, CI 95% 1.10–1.68), better reception (OR 1.33, CI 95% 1.03–1.71), better bonding and responsibility (OR 1.36, CI 95% 0.91–2.04) and better social participation (OR 1.13, CI 95% 0.93–1.35) in relation to those not regulated by primary care as the exclusive pathway of access to CEOs.

**Table 2:** Logistic regression analysis showing the coefficients of service performance in each domain by regulation of Primary Health Care, PMAQ-CEO 2018-19 study (n = 9599)

Acess	Unadjusted OR	IC 95%		Odds Ratio*	IC 95%	
Regulation	1.39	1.25	1.54	1.36	1.10	1.68
				AIC / BIC*	10.185	10.242
**Reception**						
Regulation	1.37	1.05	1.78	1.33	1.03	1.71
				AIC / BIC*	4535	4592
**Bond and responsibility**						
Regulation	1.42	0.94	2.13	1.36	0.91	2.04
				AIC / BIC*	3164	3186
**Social participation**						
Regulation	1.15	0.95	1.37	1.13	0.93	1.35
				AIC / BIC*	11.625	11.682

* Adjusted for age, sex, PHC coverage, schooling and race

## Discussion

This work presented an important main result. Regulation of CEO consultations, when carried out by primary care, showed better performance in the dimensions of access and reception. The bond and responsibility and social participation, although not significant, were better perceived by regulated users compared to those not regulated by PHC.

This study has strengths and limitations. As this is a cross-sectional study, it is not possible to make causal inferences between the associations found. As a strength, primary care was considered only when the path to CEO was carried out exclusively by scheduling appointments by the PHC, and if the health unit allowed other forms of consultation, the regulation was not considered entirely regulated. Another strong point is that the study is a representative survey of the entire national territory. As limitations, not all Dental Specialities Centers were evaluated, and it was not an obligation imposed by the PMAQ-CEO program for all health services, so we cannot generalize our findings to the country as a whole.

The Specialty Center can be characterized as a gateway to the SUS, depending on the specific characteristics of the health region in which it is located. The organization of CEOs in this parameter can harm not only the flow of referrals in the health system, but also the users of spontaneous demand, who are 4.65 times less likely to receive care from CEOs [17]. Since coordination of care is a critical challenge for primary health care, more than 36.5% of patients do not come from the consultation referred by the PHC, in agreement with Andrade et al. [2] with a result in 57.2% of the target audience [2]. Professional practice faces incompletely offered actions and care [18], which impairs the efficiency of the treatment performed by specialists.

Understood as a way of sharing health care between professionals and patients, bonding and responsibility require a demonstration of interest in the individual’s quality of life so that they feel welcomed and have an interest in continuing care, even when they return to the hospital, by counter-referencing [19]. Situations such as reduced access may reflect on patient reliability and safety, affecting the construction of the bond, which is so necessary for the acceptance and commitment [20]. In this study, 95% of the participants rated the service as good, with guidance and clarification after consultations, demonstrating a view of bonding and the favourable responsibility of the participants.

Improving existing flows in the national oral health policy guarantees better access for users to health services [18], information, and protocols for entering the SUS, an important strategy for the service cycle to work correctly [3], since direct access to speciality centers demonstrates a disarticulation between primary and specialized care, weakening counterreferral [12]. In this study, despite the lack of scheduling or an interval of more than a week for scheduling by regulation, access to the CEO was satisfactorily evaluated, with schedules that met patients’ expectations, in addition to quality in reception in the centers. Regarding the time taken to a dental consultation, most of the respondents reported that they needed less than an hour to arrive, a fact that contributes to effective access, as geographic barriers can determine a decrease in demand for care in some regions of the country [21].

The present study has results that allow us to infer that PHC, when regulating consulting for CEOs, have a better perception of two of the four dimensions of performance. However, even not statistically significant, regulation by PHC showed better bond and responsibility and social participation. According to a study by Leal et al. 2021 [22] there are still disparities in the form of access and offer of resources within the CEOs, a statement that is in line with the results of this study, where only 33.1% of the participants were asked about the best time for service. Failures in the regulation of access to CEOs may be connected to the lack of organizational controls and norms that define how referrals and counterreferrals of patients should be made, in addition to being of paramount importance that primary care professionals keep themselves updated and trained to identify cases in which regulation for specialized care is essential, as pointed out by Chaves et al. [11].

Community participation is a guideline of the SUS, established by Law 8080/1990 and institutionalized by Law 8142/1990, acting on the implementation of policies in different bodies referring to specific governmental spheres and having legal legitimacy. In a study carried out in 2020, it was found that society is not involved in health councils, a fact that creates difficulties in their consolidation [23]. The Ombudsman, an example of a democratic channel that measures the relationship between health professionals and the user, can be a resource that allows the population to contribute to the development of policies that defend the right to health [24]; however, as observed in this study, the population knows little about the resource [16]. The political discourse on social control and the existing practice of this participation, often due to the difficulty of interaction and appropriation of roles by both the State and the users, could explain both the lack of knowledge and the lack of demand for this resource found. Another important study showed that health ombudsmen have the ability to minimize racial inequalities in CEO performance [16].

Finally, it is suggested that this form of PHC regulation as the gateway for citizens and coordinators of vertical comprehensive care, will be established between the levels of oral health care in the country for better service performance.

## Data Availability

The datasets could be seen at: https://aps.saude.gov.br/ape/pmaq/ciclo2ceo/.
